# Waldenstrom's Macroglobulinemia and Peripheral Neuropathy, Organomegaly, Endocrinopathy, Monoclonal Gammopathy, and Skin Changes with a Bleeding Diathesis and Rash

**DOI:** 10.1155/2013/890864

**Published:** 2013-12-25

**Authors:** S. Haider, T. Latif, A. Hochhausler, F. Lucas, N. Abdel Karim

**Affiliations:** ^1^Department of Internal Medicine, University of Cincinnati College of Medicine, Cincinnati, OH 45267, USA; ^2^Division of Hematology and Oncology, University of Cincinnati College of Medicine, Cincinnati, OH 45267, USA; ^3^Department of Pathology, University of Cincinnati College of Medicine, Cincinnati, OH 45267, USA

## Abstract

We report a case of a 29-year-old male who presented with paraesthesia and skin lesions with excessive bleeding after skin biopsy leading to hematology consultation. He was found to have prolonged partial thromboplastin time (PTT) and monoclonal gammopathy on serum protein electrophoresis (SPEP). He experienced excessive bleeding leading to hospitalization after bone marrow biopsy and required blood transfusion. He was diagnosed with Waldenstrom's Macroglobulinemia (WM), based on the presence of IgM-**κ** type monoclonal (M) protein and infiltration of lymphoplasmacytic cells identified in bone marrow aspirates. He was noticed to have features of peripheral neuropathy, organomegaly, endocrinopathy, monoclonal gammopathy, and skin changes (POEMS syndrome). This is a very rare case of WM with POEMS syndrome which responded to chemotherapy using bortezomib, steroids, and rituximab.

## 1. Introduction

WM is characterized by the proliferation of malignant plasmacytoid lymphocytes secreting monoclonal IgM paraproteins. Incidence is three per million [1]. Usual presenting features are malaise, anemia, hemorrhage, neurological, or visual disturbances. Hyperviscosity syndrome is seen in one-third of patients [2, 3]. Our patient was unique presenting with rash and excessive bleeding after skin biopsy. Bleeding in WM is usually secondary to hyperviscosity and is an interference with clotting factor and platelet function. Chronic oozing of blood from the nose or gums is common; bleeding may occur from the gastrointestinal tract during or after surgery [4]. Rarely, WM has been associated with POEMS syndrome with three cases reported in the literature. POEMS syndrome does not typically cause a bleeding diathesis.

## 2. Case Presentation

**Figure 1:**
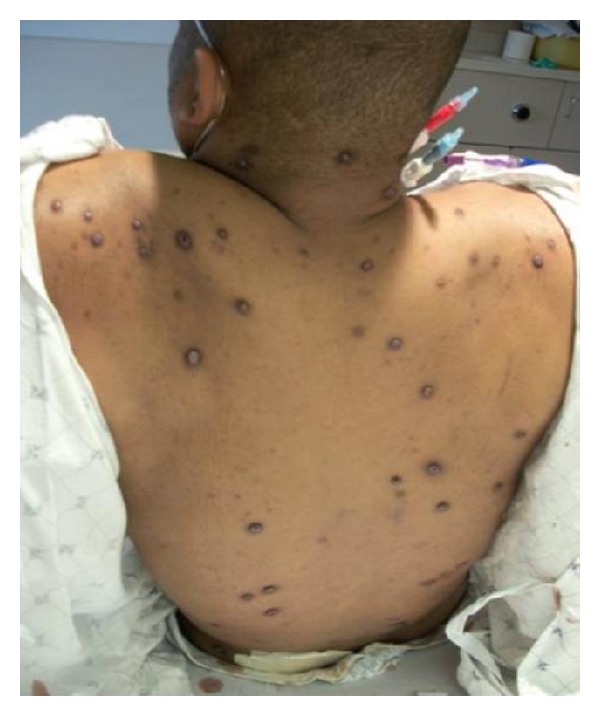
Hyperpigmented nodules with hemorrhagic crusting on the back.

**Figure 2:**
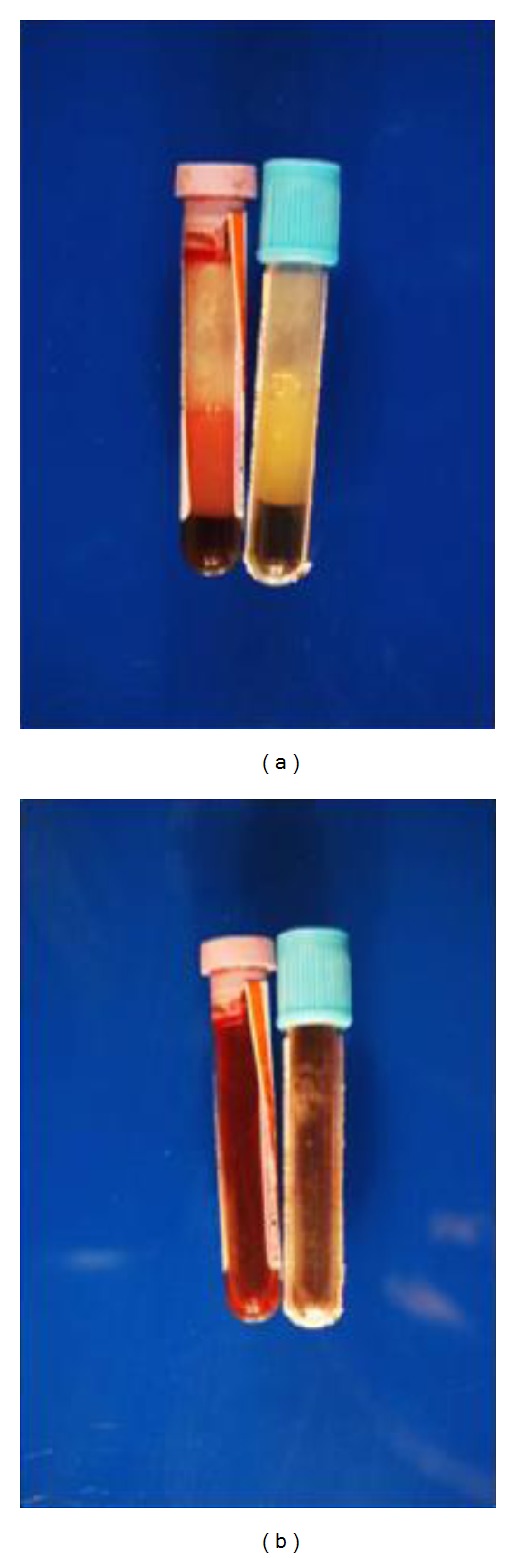
(a) EDTA and Na citrate specimens clotting at room temperature. (b) Blood specimen warmed to 37°C.

29-year-old African American male with alcohol abuse, 2-year nodular rash, shortness of breath, recurrent nosebleeds, subconjunctival hemorrhage, and paraesthesias was evaluated for excessive bleeding from skin biopsy that demonstrated congo red negative eosinophilic material. He also mentioned that he has been impotent. Physical exam was pertinent for numerous hyperpigmented nodules with hemorrhagic crusting (Figure 1), basilar crackles, dull right base, a 3/6 systolic and diastolic murmurs, impaired sensation, and paraparesis. Workup revealed a right sided pleural effusion, elevated PTT at 52.7 sec, and international normalized ratio (INR). Human immunodeficiency virus (HIV) was negative; complete blood count (CBC) and comprehensive metabolic panel (CMP) were unremarkable; SPEP revealed 4.4 g/dL M spike with kappa light chain restriction. Electrocardiogram (ECG) was unremarkable with normal voltage and intervals. Echocardiogram revealed grade 2 diastolic dysfunction, left ventricular hypertrophy, panvalvular thickening, and regurgitation. Bone survey was negative. There was mediastinal lymphadenopathy that was positron emission tomography (PET) negative. Bone marrow biopsy caused excessive bleeding and revealed kappa restricted lymphoplasmacytic infiltrate and pale eosinophilic material similar to the skin biopsy. The patient's blood samples were clotted at room temperature (Figure 2). PT/PTT was elevated and did not completely normalize with mixing; factor VII, IX, and X levels were low; serum viscosity was elevated at 3.2 centipoise (cP). The patient underwent plasma exchange for hyperviscosity when he developed symptoms of heart failure. His skin lesions, peripheral neuropathy, bleeding diathesis, and diastolic dysfunction improved with three sessions of plasma exchange and chemotherapy including steroids and bortezomib followed by rituximab.

## 3. Discussion

WM is a usually indolent lymphoplasmacytic lymphoma primarily affecting elderly white males [4, 5]. Symptoms are produced by bone marrow infiltration of tumor cells as well as the circulation, deposition, and autoantibody activity of the IgM paraproteins. The bleeding diathesis is caused by nonspecific binding of IgM to plasma proteins and cellular components. IgM can also undergo reversible precipitation at colder temperatures causing cryoglobulinemia (Figure 2). In vivo, even unprecipitated IgM can cause hyperviscosity syndrome mandating plasma exchange. The most frequent laboratory abnormality related to coagulation is prolongation of the thrombin time, a reflection of the inhibition of fibrin polymerization by the IgM paraproteins [6]. Alterations in platelet function, probably due to an interaction between the IgM paraproteins and platelet surface membrane glycoproteins, can result in prolongation of the bleeding time, impaired clot retraction, defective in vivo platelet aggregation, and decreased in vitro platelet adhesion [7]. In patients who have clinical bleeding, unexplained by hyperviscosity, the evaluation should include prothrombin time, PTT, thrombin time, and factor X activity. Hyperviscosity is an oncologic emergency and should be treated with plasmapheresis then chemotherapy to treat the underlying disease. Symptoms of hyperviscosity usually present at 4 cP at 37°C. Results can be confounded by the presence of cryoglobulins, as in this case caused by IgM precipitating at temperatures lower than 36°C. POEMS [8] is a rare disease with prevalence of 1 in 1000000. It can present in a variety of manners and has different diagnostic criteria. Mayo Clinic Criteria for the diagnosis of POEMS syndrome require the presence of at least three major criteria (polyneuropathy, monoclonal plasma cell disorder, plus any one of the following three: osteosclerotic myeloma, Castleman's disease, or elevated serum or plasma vascular endothelial growth factor (VEGF) levels at least three to four times the upper limit of normal), along with the presence of at least one of the six minor criteria organomegaly (splenomegaly, hepatomegaly, or lymphadenopathy), extravascular volume overload (edema, pleural effusion, or ascites), endocrinopathy (adrenal, thyroid, pituitary, gonadal, parathyroid, or pancreatic), skin changes (hyperpigmentation, hypertrichosis, glomeruloid hemangiomata, plethora, acrocyanosis, flushing, or white nails), papilledema, and thrombocytosis/polycythemia. Cause is unknown, likely secondary to increased VEGF [9], mostly 5- to 10-fold more than controls [10]. Plasma VEGF level >200 pg/mL had a sensitivity and specificity of 68 and 95 percent, respectively, in support of a diagnosis of POEMS syndrome. VEGF can be followed to evaluate response to therapy [11]. Patients of POEMS have higher levels of IL-1B, TNF-alpha, and IL-6 as compared to multiple myeloma. Symptoms begin as distal symmetric sensory changes in nerves of feet. The natural history is one of the progressive peripheral neuropathy until the patient is bedridden [12]. Treatments are cyclophosphamide [13] ± prednisolone; high-dose chemotherapy with stem cell transplantation [14], lenalidomide, or thalidomide; and bevacizumab [15–17].

## 4. Conclusion

WM can rarely be associated with POEMS syndrome. This is the fourth case report presenting with both WM and POEMS syndrome. Our patient presented with rash and significant bleeding, and this is the first case report to associate WM and POEMS syndrome combined, presenting with bleeding diathesis and abnormal coagulation parameters. In our case, therapy with bortezomib, rituximab, and steroids was effective after plasma exchange. Randomized studies are needed to establish the standard of care in these rare disorders.
